# Chlorido{2-[(dimethyl­amino)­meth­yl]phenyl-κ^2^ *N*,*C* ^1^}tellurium

**DOI:** 10.1107/S1600536811054328

**Published:** 2011-12-23

**Authors:** Prakul Rakesh, Harkesh B. Singh, Ray J. Butcher

**Affiliations:** a Department of Chemistry, Indian Institute of Technology Bombay, Powai, Mumbai 400 076, India; b Department of Chemistry, Howard University, 525 College Street NW, Washington, DC 20059, USA

## Abstract

The crystal structure of the title compound, C_9_H_12_ClNTe, contains isolated mol­ecules with no close Te⋯Cl inter­molecular contacts and has the same composition as a previously published structure [Engman *et al.* (2004 ▶). *Phospho­rus Sulfur Silicon Relat. Elem.* **179**, 285–292]. However, in this case, the compound has crystallized in a centrosymmetric space group, unlike the previously published structure which contained enanti­omerically pure chiral mol­ecules. In all other aspects, the metrical parameters are similar. The mol­ecules with a T-shaped coordination environment about the Te atom are linked into dimers by C—H⋯Cl inter­actions.

## Related literature

For a related structure, see: Engman *et al.* (2004 ▶). For related syntheses, see: Singh *et al.* (1990 ▶); Kaur *et al.* (1995 ▶).
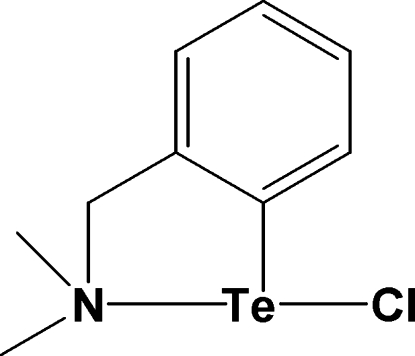


## Experimental

### 

#### Crystal data

C_9_H_12_ClNTe
*M* *_r_* = 297.25
Monoclinic, 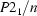

*a* = 6.4514 (6) Å
*b* = 7.0287 (7) Å
*c* = 23.847 (2) Å
β = 95.967 (9)°
*V* = 1075.49 (17) Å^3^
*Z* = 4
Mo *K*α radiation
μ = 2.96 mm^−1^
*T* = 295 K
0.45 × 0.36 × 0.12 mm

#### Data collection

Oxford Diffraction Xcalibur Ruby Gemini diffractometer
Absorption correction: multi-scan (*CrysAlis PRO*; Oxford Diffraction, 2007 ▶) *T*_min_ = 0.504, *T*_max_ = 1.000
7778 measured reflections
3587 independent reflections
2998 reflections with *I* > 2σ(*I*)
*R*_int_ = 0.023

#### Refinement

*R*[*F* ^2^ > 2σ(*F* ^2^)] = 0.043
*wR*(*F* ^2^) = 0.096
*S* = 1.20
3587 reflections
111 parameters
H-atom parameters constrained
Δρ_max_ = 2.25 e Å^−3^
Δρ_min_ = −0.98 e Å^−3^

### 

Data collection: *CrysAlis PRO* (Oxford Diffraction, 2007 ▶); cell refinement: *CrysAlis PRO*; data reduction: *CrysAlis PRO*; program(s) used to solve structure: *SHELXS97* (Sheldrick, 2008 ▶); program(s) used to refine structure: *SHELXL97* (Sheldrick, 2008 ▶); molecular graphics: *XP* in *SHELXTL* (Sheldrick, 2008 ▶); software used to prepare material for publication: *SHELXTL*.

## Supplementary Material

Crystal structure: contains datablock(s) I, global. DOI: 10.1107/S1600536811054328/pk2376sup1.cif

Structure factors: contains datablock(s) I. DOI: 10.1107/S1600536811054328/pk2376Isup2.hkl

Additional supplementary materials:  crystallographic information; 3D view; checkCIF report

## Figures and Tables

**Table 1:** Selected bond lengths (Å)

Te—C1	2.116 (3)
Te—N	2.355 (3)
Te—Cl	2.5657 (11)

**Table 2:** Hydrogen-bond geometry (Å, °)

*D*—H⋯*A*	*D*—H	H⋯*A*	*D*⋯*A*	*D*—H⋯*A*
C9—H9*C*⋯Cl^i^	0.96	2.89	3.822 (5)	163

Symmetry code: (i) 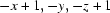.

**Fig. 1.**
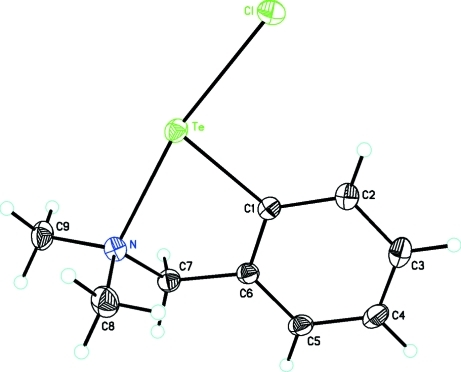
Diagram of the contents of the asymmetric unit of C9H12ClNTe. Atomic displacement ellipsoids are drawn at the 30% level.

**Fig. 2.**
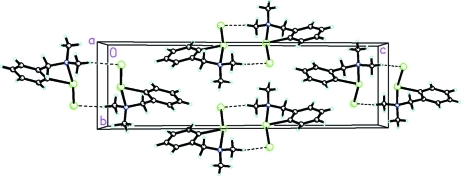
The molecular packing for C9H12ClNTe viewed down the c axis. C—H···Cl secondary interactions are shown by dashed lines.

## supplementary crystallographic information

### Comment

Attempts to synthesize protonated bis[2-(dimethylaminomethyl)phenyl]ditelluride resulted in the formation of 2-(*N*,*N*-dimethylaminomethyl)phenyl)tellurenyl chloride, C_9_H_12_ClNTe, (1) a compound which had been previously prepared *via* a different method (Singh *et al.*, 1990).  The structure of C_9_H_12_ClNTe contains isolated molecules with no close Te···Cl intermolecular contacts, and is chemically related to a previously published structure (Engman, *et al.*, 2004), even though it had been prepared by the same method as the title compound.  However, in this case the compound has crystallized in a centrosymmetric space group, unlike the previously published structure (Engman, *et al.*, 2004) which contained enantiomerically pure, chiral molecules. In all other aspects the metrical parameters are similar. The molecules arelinked into dimers by C—H···Cl interactions.

### Experimental

A stirred solution of bis[2-(dimethylaminomethyl)phenyl]ditelluride (Kaur *et al.*, 1995) (0.5 g, 0.94 mmol) in diethylether (10 ml) was treated with HCl (3 ml in 20 ml distilled water). The reaction mixture was further stirred for 10 min. The resulting reaction mixture was evaporated to one third of its original volume and ethanol (5 ml) was added to get a yellow solid. It was redissolved in ethanol and stored in the refrigerator to get yellow needles of X-ray quality crystals. Yield 0.2 g, 35% mp 121°C (lit value 121°C). Anal. Calcd for C_9_H_12_ClTe: C, 36.37; H, 4.07; N, 4.37. Found C, 36.44; H, 4.04; N, 4.66.

### Refinement

H atoms were placed in geometrically idealized positions and constrained to ride on their parent atoms with C—H distances of 0.95 - 0.97 Å [*U*_iso_(H) = 1.2*U*_eq_(CH, CH_2_) [*U*_iso_(H) = 1.5*U*_eq_(CH_3_)].

### Crystal data

**Table:**

C_9_H_12_ClNTe	*F*(000) = 568
*M**_r_* = 297.25	*D*_x_ = 1.836 Mg m^−^^3^
Monoclinic, *P*2_1_/*n*	Mo *K*α radiation, λ = 0.71073 Å
Hall symbol: -P 2yn	Cell parameters from 3931 reflections
*a* = 6.4514 (6) Å	θ = 5.1–32.6°
*b* = 7.0287 (7) Å	µ = 2.96 mm^−^^1^
*c* = 23.847 (2) Å	*T* = 295 K
β = 95.967 (9)°	Irregular plate, pale yellow
*V* = 1075.49 (17) Å^3^	0.45 × 0.36 × 0.12 mm
*Z* = 4	

### Data collection

**Table:**

Oxford Diffraction Xcalibur Ruby Gemini diffractometer	3587 independent reflections
Radiation source: fine-focus sealed tube	2998 reflections with *I* > 2σ(*I*)
graphite	*R*_int_ = 0.023
Detector resolution: 10.5081 pixels mm^-1^	θ_max_ = 32.7°, θ_min_ = 5.1°
ω scans	*h* = −9→9
Absorption correction: multi-scan (*CrysAlis PRO*; Oxford Diffraction, 2007)	*k* = −6→10
*T*_min_ = 0.504, *T*_max_ = 1.000	*l* = −35→34
7778 measured reflections	

### Refinement

**Table:**

Refinement on *F*^2^	Primary atom site location: structure-invariant direct methods
Least-squares matrix: full	Secondary atom site location: difference Fourier map
*R*[*F*^2^ > 2σ(*F*^2^)] = 0.043	Hydrogen site location: inferred from neighbouring sites
*wR*(*F*^2^) = 0.096	H-atom parameters constrained
*S* = 1.20	*w* = 1/[σ^2^(*F*_o_^2^) + (0.0327*P*)^2^ + 1.2847*P*] where *P* = (*F*_o_^2^ + 2*F*_c_^2^)/3
3587 reflections	(Δ/σ)_max_ = 0.001
111 parameters	Δρ_max_ = 2.25 e Å^−^^3^
0 restraints	Δρ_min_ = −0.98 e Å^−^^3^

### Special details

**Table:**

Geometry. All e.s.d.'s (except the e.s.d. in the dihedral angle between two l.s. planes) are estimated using the full covariance matrix. The cell e.s.d.'s are taken into account individually in the estimation of e.s.d.'s in distances, angles and torsion angles; correlations between e.s.d.'s in cell parameters are only used when they are defined by crystal symmetry. An approximate (isotropic) treatment of cell e.s.d.'s is used for estimating e.s.d.'s involving l.s. planes.
Refinement. Refinement of *F*^2^ against ALL reflections. The weighted *R*-factor *wR* and goodness of fit *S* are based on *F*^2^, conventional *R*-factors *R* are based on *F*, with *F* set to zero for negative *F*^2^. The threshold expression of *F*^2^ > σ(*F*^2^) is used only for calculating *R*-factors(gt) *etc*. and is not relevant to the choice of reflections for refinement. *R*-factors based on *F*^2^ are statistically about twice as large as those based on *F*, and *R*- factors based on ALL data will be even larger.

### Fractional atomic coordinates and isotropic or equivalent isotropic displacement parameters (Å^2^)

**Table:**

	*x*	*y*	*z*	*U*_iso_*/*U*_eq_	
Te	0.58681 (4)	0.01772 (4)	0.423777 (10)	0.03896 (9)	
Cl	0.29601 (17)	−0.23269 (17)	0.41307 (5)	0.0537 (3)	
N	0.8374 (5)	0.2560 (4)	0.41417 (13)	0.0372 (6)	
C1	0.5512 (6)	0.0787 (5)	0.33640 (14)	0.0347 (7)	
C2	0.3769 (6)	0.0346 (6)	0.29976 (16)	0.0413 (8)	
H2A	0.2649	−0.0291	0.3127	0.050*	
C3	0.3712 (7)	0.0865 (7)	0.24370 (18)	0.0505 (10)	
H3A	0.2543	0.0571	0.2190	0.061*	
C4	0.5367 (8)	0.1816 (6)	0.22353 (17)	0.0515 (10)	
H4A	0.5311	0.2154	0.1857	0.062*	
C5	0.7099 (7)	0.2255 (5)	0.26028 (16)	0.0456 (9)	
H5A	0.8212	0.2899	0.2471	0.055*	
C6	0.7191 (6)	0.1741 (5)	0.31665 (15)	0.0366 (7)	
C7	0.9058 (6)	0.2144 (6)	0.35786 (15)	0.0416 (8)	
H7A	0.9982	0.1052	0.3604	0.050*	
H7B	0.9816	0.3225	0.3450	0.050*	
C8	0.7367 (8)	0.4460 (6)	0.4157 (2)	0.0588 (12)	
H8A	0.8373	0.5433	0.4104	0.088*	
H8B	0.6837	0.4636	0.4515	0.088*	
H8C	0.6240	0.4539	0.3861	0.088*	
C9	1.0127 (6)	0.2409 (7)	0.45859 (18)	0.0538 (11)	
H9A	1.1119	0.3397	0.4537	0.081*	
H9B	1.0786	0.1191	0.4562	0.081*	
H9C	0.9620	0.2539	0.4948	0.081*	

### Atomic displacement parameters (Å^2^)

**Table:**

	*U*^11^	*U*^22^	*U*^33^	*U*^12^	*U*^13^	*U*^23^
Te	0.03675 (13)	0.04765 (15)	0.03311 (13)	0.00064 (11)	0.00659 (8)	0.00140 (11)
Cl	0.0484 (5)	0.0601 (6)	0.0536 (6)	−0.0112 (5)	0.0102 (4)	0.0036 (5)
N	0.0340 (14)	0.0375 (15)	0.0403 (16)	0.0027 (12)	0.0043 (12)	−0.0043 (13)
C1	0.0397 (17)	0.0331 (15)	0.0320 (16)	0.0061 (14)	0.0069 (13)	−0.0021 (13)
C2	0.0409 (18)	0.0428 (19)	0.0399 (19)	0.0057 (16)	0.0039 (14)	−0.0059 (16)
C3	0.057 (2)	0.050 (2)	0.042 (2)	0.014 (2)	−0.0078 (18)	−0.0079 (18)
C4	0.079 (3)	0.039 (2)	0.0348 (19)	0.010 (2)	0.0016 (19)	0.0035 (16)
C5	0.066 (3)	0.0367 (18)	0.0357 (19)	−0.0026 (18)	0.0112 (17)	0.0009 (15)
C6	0.0438 (18)	0.0320 (16)	0.0348 (17)	−0.0002 (14)	0.0071 (14)	−0.0014 (14)
C7	0.0404 (18)	0.049 (2)	0.0364 (18)	−0.0033 (16)	0.0097 (14)	−0.0003 (16)
C8	0.071 (3)	0.043 (2)	0.061 (3)	0.007 (2)	0.000 (2)	−0.012 (2)
C9	0.042 (2)	0.074 (3)	0.043 (2)	−0.007 (2)	−0.0038 (17)	−0.004 (2)

### Geometric parameters (Å, °)

**Table:**

Te—C1	2.116 (3)	C4—H4A	0.9300
Te—N	2.355 (3)	C5—C6	1.387 (5)
Te—Cl	2.5657 (11)	C5—H5A	0.9300
N—C9	1.471 (5)	C6—C7	1.500 (5)
N—C7	1.486 (5)	C7—H7A	0.9700
N—C8	1.487 (5)	C7—H7B	0.9700
C1—C2	1.385 (5)	C8—H8A	0.9600
C1—C6	1.397 (5)	C8—H8B	0.9600
C2—C3	1.383 (6)	C8—H8C	0.9600
C2—H2A	0.9300	C9—H9A	0.9600
C3—C4	1.387 (7)	C9—H9B	0.9600
C3—H3A	0.9300	C9—H9C	0.9600
C4—C5	1.382 (6)		
			
C1—Te—N	76.45 (13)	C6—C5—H5A	119.8
C1—Te—Cl	92.14 (10)	C5—C6—C1	119.7 (4)
N—Te—Cl	168.59 (8)	C5—C6—C7	122.4 (3)
C9—N—C7	111.0 (3)	C1—C6—C7	117.9 (3)
C9—N—C8	110.7 (3)	N—C7—C6	109.6 (3)
C7—N—C8	111.7 (3)	N—C7—H7A	109.7
C9—N—Te	111.3 (3)	C6—C7—H7A	109.7
C7—N—Te	102.7 (2)	N—C7—H7B	109.7
C8—N—Te	109.3 (3)	C6—C7—H7B	109.7
C2—C1—C6	120.1 (3)	H7A—C7—H7B	108.2
C2—C1—Te	125.0 (3)	N—C8—H8A	109.5
C6—C1—Te	114.9 (3)	N—C8—H8B	109.5
C3—C2—C1	119.3 (4)	H8A—C8—H8B	109.5
C3—C2—H2A	120.4	N—C8—H8C	109.5
C1—C2—H2A	120.4	H8A—C8—H8C	109.5
C2—C3—C4	121.2 (4)	H8B—C8—H8C	109.5
C2—C3—H3A	119.4	N—C9—H9A	109.5
C4—C3—H3A	119.4	N—C9—H9B	109.5
C5—C4—C3	119.3 (4)	H9A—C9—H9B	109.5
C5—C4—H4A	120.4	N—C9—H9C	109.5
C3—C4—H4A	120.4	H9A—C9—H9C	109.5
C4—C5—C6	120.5 (4)	H9B—C9—H9C	109.5
C4—C5—H5A	119.8		
			
C1—Te—N—C9	−152.7 (3)	C2—C3—C4—C5	−0.2 (6)
Cl—Te—N—C9	−154.3 (3)	C3—C4—C5—C6	0.4 (6)
C1—Te—N—C7	−33.9 (2)	C4—C5—C6—C1	−0.5 (6)
Cl—Te—N—C7	−35.5 (5)	C4—C5—C6—C7	178.5 (4)
C1—Te—N—C8	84.8 (3)	C2—C1—C6—C5	0.3 (5)
Cl—Te—N—C8	83.2 (5)	Te—C1—C6—C5	−178.4 (3)
N—Te—C1—C2	−160.8 (3)	C2—C1—C6—C7	−178.7 (3)
Cl—Te—C1—C2	18.9 (3)	Te—C1—C6—C7	2.6 (4)
N—Te—C1—C6	17.8 (2)	C9—N—C7—C6	163.4 (3)
Cl—Te—C1—C6	−162.5 (3)	C8—N—C7—C6	−72.6 (4)
C6—C1—C2—C3	−0.1 (5)	Te—N—C7—C6	44.4 (3)
Te—C1—C2—C3	178.4 (3)	C5—C6—C7—N	145.9 (3)
C1—C2—C3—C4	0.0 (6)	C1—C6—C7—N	−35.1 (5)

### Hydrogen-bond geometry (Å, °)

**Table:**

*D*—H···*A*	*D*—H	H···*A*	*D*···*A*	*D*—H···*A*
C9—H9C···Cl^i^	0.96	2.89	3.822 (5)	163.

Symmetry codes: (i) −*x*+1, −*y*, −*z*+1.
